# Formation of 3-Dimensional Gold, Copper and Palladium Microelectrode Arrays for Enhanced Electrochemical Sensing Applications

**DOI:** 10.3390/nano9081170

**Published:** 2019-08-15

**Authors:** Catherine E. Hay, Junqiao Lee, Debbie S. Silvester

**Affiliations:** Curtin Institute for Functional Molecules and Interfaces, School of Molecular and Life Sciences, Curtin University, GPO Box U1987, Perth 6845, WA, Australia

**Keywords:** 3D nanostructures, microarrays, electrodeposition, gold, copper, palladium, TNT, carbon dioxide, hydrogen, room-temperature ionic liquids

## Abstract

Microelectrodes offer higher current density and lower ohmic drop due to increased radial diffusion. They are beneficial for electroanalytical applications, particularly for the detection of analytes at trace concentrations. Microelectrodes can be fabricated as arrays to improve the current response, but are presently only commercially available with gold or platinum electrode surfaces, thus limiting the sensing of analytes that are more electroactive on other surfaces. In this work, gold (Au), copper (Cu), and palladium (Pd) are electrodeposited at two different potentials into the recessed holes of commercial microelectrode arrays to produce 3-dimensional (3D) spiky, dendritic or coral-like structures. The rough fractal structures that are produced afford enhanced electroactive surface area and increased radial diffusion due to the 3D nature, which drastically improves the sensitivity. 2,4,6-trinitrotoluene (TNT), carbon dioxide gas (CO_2_), and hydrogen gas (H_2_) were chosen as model analytes in room temperature ionic liquid solvents, to demonstrate improvements in the sensitivity of the modified microelectrode arrays, and, in some cases (e.g., for CO_2_ and H_2_), enhancements in the electrocatalytic ability. With the deposition of different materials, we have demonstrated enhanced sensitivity and electrocatalytic behaviour towards the chosen analytes.

## 1. Introduction

Electrochemical (amperometric) gas sensors are commonly used to detect chemical species and are widely commercially available from a number of companies [1]. Amperometric sensors offer several advantages compared to other analytical techniques, including low power consumption, the ability to operate efficiently at room temperature, simple design and fabrication, high portability, and low cost. They are often used for agricultural, clinical, environmental, workplace safety and industrial analysis [1]. They work by employing an applied potential between the counter and working electrode, resulting in a current that is proportional to analyte concentration [2,3,4]. In order to expand the applications of such devices, a focus has been placed on their miniaturisation, particularly with the recent availability of low-cost planar electrode devices [5,6]. Thin-film electrodes (TFEs) are one type of planar electrode device, consisting of three electrodes—working, reference and counter—integrated onto a single platform in a small area [7]. Macro- and micro-electrode designs are available from commercial suppliers, and they are typically made from only a thin layer (~150 nm) of noble metal electrode material, thus significantly reducing costs compared to conventional disk electrodes.

**Figure 1:**
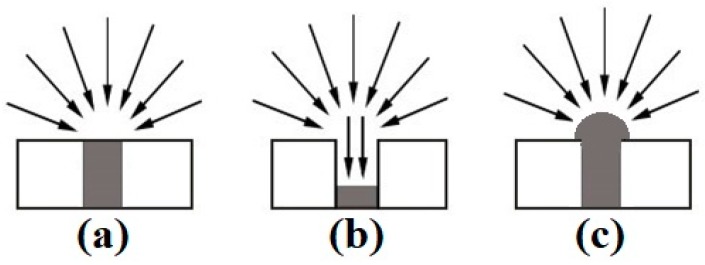
Illustration of diffusion profiles to (**a**) an inlaid disc, (**b**) a recessed disc, and (**c**) a hemispherical microelectrode [10].

Micro-electrodes are particularly beneficial for electroanalytical applications due to the high current density and lower ohmic drop as a result of increased radial diffusion (see Figure 1a) [8,9]. However, the smaller electrode size results in a reduction in the electroactive surface area, which reduces the overall current response. To combat this, arrays of microdisks have been developed to increase the electroactive surface area. The microarray thin-film electrodes (MATFEs) used in this work contain 90 recessed electrodes of 10 µm in diameter, evenly spaced and sufficiently separated to ensure that their diffusion profiles do not overlap [10]. The very small size of the electrodes enables tiny volumes (e.g., 1–5 µL) of non-volatile room-temperature ionic liquids (RTILs) to be used as solvents. However, the drawback to the commercial MATFE design is that the electrodes are recessed within a SU-8 polymer layer. This results in reduced current density due to contributions from linear diffusion within the hole (see Figure 1b) [10,11].

In order to overcome the lower current density of the recessed electrodes, Hussain et al. [12,13] created three-dimensional (3D) Pt nanostructures by electrodeposition, using the holes of the recessed arrays as templates for the growth, in an attempt to form hemispherical electrodes (see Figure 1c). When the hole was filled with Pt metal to create an almost inlaid electrode (Figure 1a), the sensitivity towards ammonia gas in RTILs increased by 42% [14]. However, a 6.5-times sensitivity increase towards ammonia was observed when the holes were overfilled to form 3D cauliflower-like structures [12]. Furthermore, the addition of lead acetate to the electrodeposition bath resulted in the formation of dendritic structures with even higher surface areas [13]. It was found that depositing in two separate steps produced the most spiky 3-dimensional structures, significantly increasing the electroactive surface area of the electrode compared to the more rounded cauliflower deposits, with a 16-times increase in current for the oxygen reduction observed [13]. Electrodeposited metals are presumed to be more robust than those formed using other methods (e.g., chemical vapour deposition (CVD) and physical vapour deposition (PVD)) since they are rigidly attached to the underlying electrode surface [15]. Electrodeposition presents a wide range of controllable parameters to tailor the deposition to the requirements of the sensor, and the technique is also relatively simple to perform, low cost and requires a low power consumption, particularly compared to CVD and PVD methods.

Presently, MATFEs are only commercially available with Au and Pt electrode materials, which limits sensing applications for species that have greater electroactivity on other surfaces. Previous work on the modification of MATFEs has been limited to Pt deposition on Pt substrates [12,13,14]. By expanding the deposition to different metals, we demonstrate a simple, inexpensive method to selectively target different analytes. Therefore, in this study, we have investigated the electrodeposition of Au, Cu and Pd at both higher (over-) and lower (under-)potentials, where at higher potentials, the growth of the structure is diffusion-limited, whereas at the lower potentials, it is kinetically controlled [12,14]. We not only observe the structural differences as the result of different nucleation and growth mechanisms, but also show how the different growths affect the sensing current. Room temperature ionic liquids (RTILs) are used as solvents to determine the sensitivity enhancements towards three chosen analytes, 2,4,6-trinitrotoluene (TNT), carbon dioxide (CO_2_) and hydrogen (H_2_). By electrodepositing different metals into the holes of the MATFEs, we demonstrate a method to extend the type of materials assessable, to selectively target sensing towards different analytes of interest.

## 2. Materials and Methods

### 2.1. Chemical Reagents

Tetrachloroauric(III) acid hydrate (HAuCl_4_.xH_2_O, trace metal basis, ≥99.9%, Merck, Kilsyth, VIC, Australia), copper(II) nitrate hemi(pentahydrate) (Cu(NO_3_)_2_·2.5H_2_O, 98%, Chem-Supply, Gillman, SA Australia), palladium(II) chloride (PdCl_2_, 99%, Merck, Kilsyth, VIC, Australia) and sulfuric acid (H_2_SO_4_, 98% *w*/*w*, Merck, Kilsyth, VIC, Australia) were used as received. The room temperature ionic liquids (RTILs) 1-butyl-3-methylimidazolium bis(trifluoromethylsulfonyl)imide ([C_4_mim] [NTf_2_], >99%) and *N*-butyl-*N*-methylpyrrolidinium bis(trifluoromethylsulfonyl)imide ([C_4_mpyrr] [NTf_2_], >99%) were purchased from Merck (Kilsyth, VIC, Australia), at ultra-high purity electrochemical grade. All chemicals were used as received without any further purification. Ultrapure water with a resistance of 18.2 MΩ cm was prepared by an ultrapure water purification system (Millipore Pty Ltd., North Ryde, NSW, Australia). Carbon dioxide (99.9%) was purchased from Coregas Pty. Ltd. (Jandakot, WA, Australia). Hydrogen and nitrogen (≥99.99%) gases were from BOC gases (Welshpool, WA, Australia). 2,4,6-Trinitrotoluene (TNT, 1000 µg/1 mL in acetonitrile) was from Cerilliant Corporation, Round Rock, TX, USA.

### 2.2. Electrochemical Experiments

**Figure 2:**
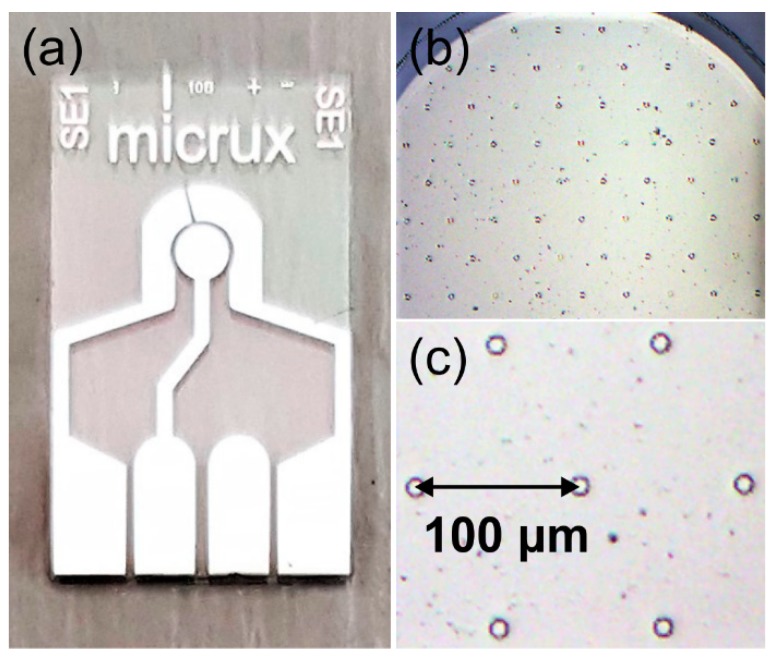
(**a**) Photo of a platinum MicruX microarray thin-film electrode, (**b**) optical microscope image of the microarray working electrode defined across an SU-8 overlayer, and (**c**) further zoomed-in optical microscope image showing the pitch distance between two µ-holes.

All experiments were performed using a PGSTAT101 Autolab potentiostat (Eco Chemie, Utrecht, The Netherlands) interfaced to a PC with Nova 1.11 software, at laboratory room temperature (294 ± 1 K) inside an aluminum Faraday cage (to reduce electrical interference), and situated within a fume cupboard. Platinum (Pt, ED-mSE-10-Pt) and gold (Au, ED-mSE-10-Au) microarray thin-film electrodes (MATFEs, Micrux Technologies, Oviedo, Spain), consisted of 90 recessed µ-holes of 10 µm diameter (defined by a SU-8 layer on a 1 mm diameter working electrode) in a hexagonal arrangement, with a pitch distance of 100 ± 1 µm (10× diameter) and depth of 3.5 ± 1.0 µm. The Pt thin-film counter and quasi-reference electrodes were in close proximity to the working electrode. A photograph of the electrode, and close-up optical microscope images of the microarrays are shown in Figure 2.

#### 2.2.1. Electrochemical Deposition of Metal Nanostructures

The MATFEs were electrochemically activated in 0.5 M H_2_SO_4(aq)_ by scanning the potential between −0.4 and +1.4 V vs. an external Ag/AgCl (1 M KCl (aq)) reference electrode (BASi, West Lafayette, IN, USA) and a Pt coil counter electrode at a sweep rate of 500 mV·s^−1^ for ca. 300 cycles. The electrodes were then washed in ultrapure water and dried under a nitrogen stream.

The recessed micro-holes were used as templates during the electrodeposition of Au, Cu and Pd metals to form deposits with different shapes and sizes. The Au plating solution consisted of 20 mM HAuCl_4_ in 0.5 M H_2_SO_4(aq)_. Starting at the open circuit potential (OCP, ~0.8 V), a higher negative potential (termed ‘overpotential’ in this work) (0.1 V) or a lower negative potential (termed ‘underpotential’ in this work) (0.5 V) vs. an external Ag/AgCl reference electrode was applied, while limiting the total charge of the deposition procedure to −1.5 mC. For the deposition of copper, a bath of 20 mM Cu(NO_3_)_2_ was employed with 0.5 M NaNO_3(aq)_. From the open circuit potential (OCP, ~0.5V) an ‘overpotential’ (−0.4 V) or an ‘underpotential’ (−0.2 V) vs. an external Ag/AgCl was applied, while limiting the total charge to −4.5 mC. Palladium deposition was performed from a 20 mM solution of PdCl_2_ in 0.5 M H_2_SO_4_. From the open circuit potential (OCP, ~0.6V), an ‘overpotential’ (0.1 V) or an ‘underpotential’ (0.35 V) vs. an external Ag/AgCl was applied, while limiting the total charge to −1.5 mC. For all depositions, the rate of mass transport was fixed by using a fast rate of stirring with a magnetic stirrer. Different charge limits for each metal were selected to ensure a similar sized deposit was obtained, confirmed by analysing scanning electron microscopy images. The Au and Cu structures produced via electrodeposition appeared to be strongly attached to the substrate, and there were no signs of detachment even with vigorous rinsing and subsequent drying under a strong air flow. In contrast, more care was required with the handling of the Pd electrodes, as segments of the structures readily detached during washing.

MATFEs modified with electrodeposited Au and Pd (hereafter referred to as Au modWEs and Pd modWEs, respectively) were electrochemically activated in 0.5 M H_2_SO_4(aq)_ by scanning the potential between +1.40 V and −0.4 V vs. Ag/AgCl (1 M KCl) reference electrode and a Pt coil counter electrode, at a sweep rate of 500 mVs^−1^ for ca. 10 cycles before being utilised in the respective sensing experiments. Cu-modified MATFEs (Cu ModWEs) were used directly after deposition, due to the instability of the Cu deposits in acidic solutions.

#### 2.2.2. Electrochemical Sensing Experiments in Ionic Liquids

For the detection of 2,4,6-trinitrotoluene (TNT), 1–10 mM solutions of TNT were prepared using appropriate volumes of TNT (in acetonitrile from 1000 µg/1 mL stock solution). The acetonitrile was left to evaporate overnight in a vacuum desiccator, before the addition of [C_4_mim] [NTf_2_]. After the TNT was fully dissolved, 5 µL of the TNT/RTIL mixture was dropcast on the electrodes. The electrodes were held with a modified rubber stopper housed inside a glass cell and purged under nitrogen gas for at least 30 min to remove dissolved gases and impurities in the ionic liquid.

The gas-sensing setup is illustrated in detail in the supplementary information of our previous paper [16]. Briefly, 3 µL of RTIL was dropcasted onto the MATFE, and was placed into an airtight glass cell. Prior to the introduction of the analyte gas, the cell was purged with nitrogen for >30 min to remove dissolved gases and impurities. To obtain different concentrations, the analyte gas was diluted with nitrogen gas using two digital flow meters (0–1.0 L/min, John Morris Scientific, Chatswood, NSW, Australia), one connected to the analyte gas cylinder and the other the nitrogen cylinder through PTFE tubing via a Swagelok T-joint (Swagelok, Kardinya, WA, Australia). The relative flow rates of the gases were used to calculate the concentration of analyte gas introduced into the cell. The cell was allowed to equilibrate for 20 min at each concentration before proceeding with voltammetric scans.

### 2.3. Electrode Imaging

Scanning electron microscopy (SEM) was performed on the working electrodes of the deposited MATFEs both prior to and after electrochemical sensing experiments. Images were acquired using the Neon Dual-Beam FESEM (Ziess, Neon 40EsB; Oberkochen, Germany) instrument at the John de Laeter Centre at Curtin University.

## 3. Results and Discussion

### 3.1. Electrochemical Deposition of 3D Nanostructured Microarrays

**Figure 3:**
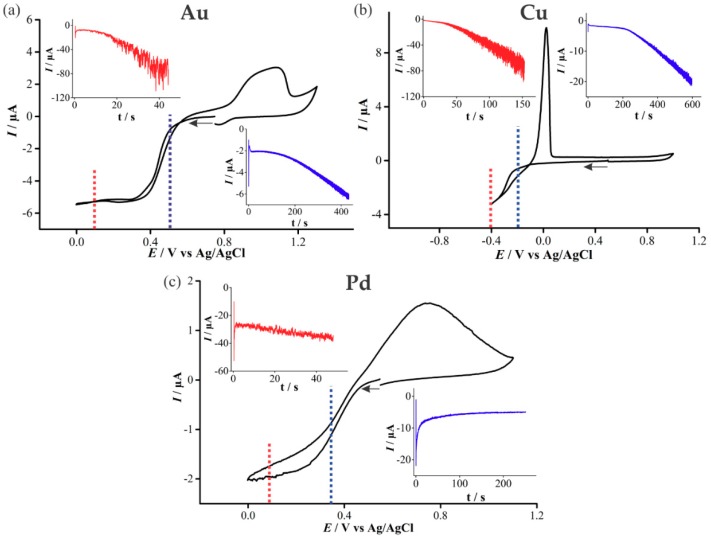
Cyclic voltammetry (CV) at 100 mVs^−1^ on a recessed Pt microarray thin-film electrode (MATFE) (90 holes, 10 µm in diameter) in (**a**) 20 mM N_2_-saturated HAuCl_4_ in 0.5 M H_2_SO_4_, (**b**) 20 mM N_2_-saturated Cu(NO_3_)_2_ in 0.5 M NaNO_3_, (**c**) 20 mM N_2_-saturated PdCl_2_ in 0.5 M H_2_SO_4_. The insets show the chronoamperometric transients for the deposition of Au, Pd and Cu at an overpotential and an underpotential into the microholes, held at the potentials indicated by the dotted lines in the CV.

In order to determine appropriate potentials for the electrodeposition of Au, Cu, and Pd, cyclic voltammetry (CV) was performed in 20 mM solutions of the precursor metal salts (Figure 3). At higher (over)-potentials, progressive nucleation is expected to form rougher deposits, whereas in the kinetically controlled region at lower (under)-potentials, instantaneous nucleation and smoother deposits are expected [17]. Both overpotential and underpotential deposition will be performed for all three metals in this study.

For the deposition of Au (Figure 3a), a single reduction feature is present, beginning at ~0.5 V, consistent with previous studies [18,19], and is attributed to the reduction of AuCl_4_^−^_(aq)_ to metallic gold:

(1) $${\left[{\mathrm{AuCl}}_{4}\right]}^{-}+3e^{-}\to \;\mathrm{Au}+4{\mathrm{Cl}}^{-}$$

The reduction wave exhibits steady-state behaviour due to the predominant radial diffusion process to the micro-holes. Current crossovers occur at 0.08 V and 0.57 V, where the current for the oxidation sweep remains below the original reduction sweep, indicative of a nucleation and growth mechanism occurring [20,21]. An oxidation feature is present at potentials more positive than 0.8 V, and the broad nature of this peak suggests it corresponds to multiple processes, probably the electro-dissolution of metallic gold, as well as the formation of an oxide layer on the electrodeposited gold [19].

The upper inset in Figure 3a shows the chronoamperometric transient obtained for the electrodeposition of Au at an overpotential (0.1 V). For the first 20 s, the current increases gradually, after which it begins to increase more substantially with large current oscillations, corresponding to the growths forming over the edges of the micro-holes. This is consistent with our previous study on platinum deposition [12]. The lower inset in Figure 3a shows the chronoamperometric transient for underpotential (0.5 V) deposition of Au. An increase in current magnitude and larger oscillations occur above ~200 s, akin to that of the overpotential deposition case. However, the current magnitude is significantly smaller, and the transient has to be measured for 450 s, compared to 50 s for the overpotential deposition, to pass the same amount of charge.

For the deposition of Cu, the reduction sweep on the voltammogram (Figure 3b) shows a broad feature starting at ~−0.15 V, described by the following equation [22,23]:

(2) $${\mathrm{Cu}}^{2+}+2e^{-}\to \;\mathrm{Cu}$$

As with Au, a current crossover occurs on the reverse sweep due to Cu nucleation and the formation of a different material on the electrode surface [19]. Furthermore, a large oxidative peak on the reverse sweep is visible, demonstrating the ease of “stripping” the metal off the surface. The increasing current oscillations in the chronoamperometric transient for Cu deposition was similar to that of Au deposition. However, a greater charge and a longer length of time was required to form a similar sized deposit due to the different nature of the material. The Cu overpotential amperometric deposition reaches a similar transient current as the Au overpotential deposition current. However, the underpotential deposition reaches more than double the current magnitude of its Au counterpart.

Palladium(II) and chloride ions can form four types of complexes in acidic media with PdCl_4_^2−^ being the most stable species [24]. The reduction feature observed at 0.37 V in Figure 3c is due to the reduction of Pd according to the following equation:

(3) $${\mathrm{PdCl}}_{4}{}^{2-}+2e^{-}\to \;\mathrm{Pd}+4{\mathrm{Cl}}^{-}$$

The anodic peak at 0.7 V is attributed to the dissolution of deposited palladium and oxide formation [24]. The chronoamperometric behavior during Pd deposition is quite different to the other metals in this study, and with Pt in our previous study [12]. The upper inset in Figure 3c shows uniform current oscillations throughout, as well as only a slight increase in current magnitude as the deposition proceeds. The underpotential deposition shows a significant change in the behaviour compared to Au, Cu and previous studies of Pt [12], displaying instead a decrease in current that approaches a steady value. This underpotential deposition transient behaviour was found to be reproducible on several electrodes.

### 3.2. Characterisation of 3D Nanostructured Microarrays

**Figure 4:**
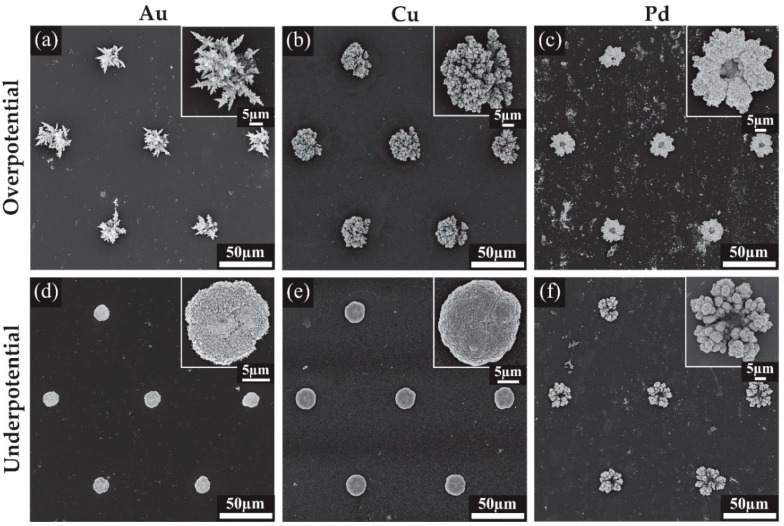
Scanning electron microscopy (SEM) images for the 3-dimensional structures formed on Pt MATFEs using Au, Cu and Pd with insets of a magnified single structure. (**a**) Au at 0.1 V, (**b**) Cu at −0.4 V, (**c**) Pd at 0.1 V, (**d**) Au at 0.5 V, (**e**) Cu at −0.2 V, (**f**) Pd at 0.35 V.

**Figure 5:**
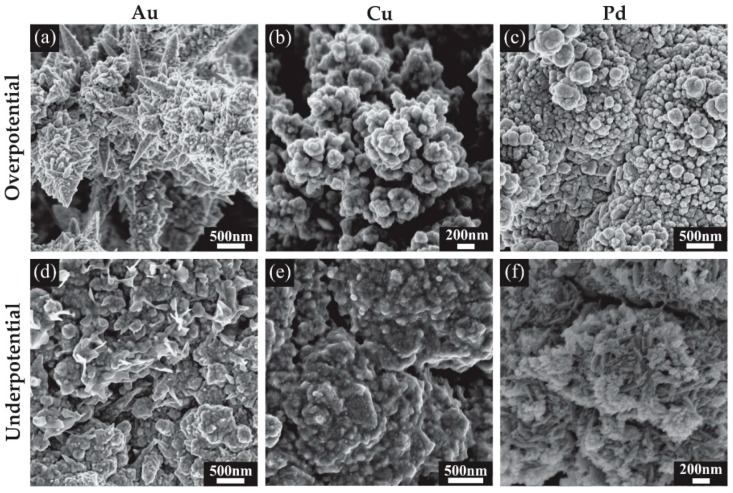
Scanning electron microscopy (SEM) images showing the surface detail on the electrodeposited nanostructures. Deposition parameters: (**a**) Au at 0.1 V, (**b**) Cu at −0.4 V, (**c**) Pd at 0.1 V, (**d**) Au at 0.5 V, (**e**) Cu at −0.2 V, (**f**) Pd at 0.35 V.

Scanning electron microscopy (SEM) was used to visualise the deposited structures formed at different deposition potentials. Figure 4 shows the SEM images for all the modified surfaces, while Figure 5 shows close-up images to reveal the fine structure of the deposits. Figure 4a shows an array of Au microstructures formed at an overpotential of 0.1 V with the charge limited to −1.5 mC. This deposition results in large spiky dendritic structures as shown in the inset of Figure 4a. The further zoomed-in images in Figure 5a reveal thorn-like protrusions along each of the dendritic branches. Typically, without known bath additions such as Pb(OAc)_2_ or cysteine, such dendritic structures are not expected to be formed [25,26,27]. The Au deposits from an underpotential (0.5 V) deposition are shown in Figure 4d. The inset shows a close-up image of a dense structure with a smoother appearance and no significant dendrite formation. Further magnification in Figure 5d reveals a rough surface with some spiky protrusions distributed on the deposit, increasing the surface area compared to a smooth surface. Furthermore, the 3-D nature of the deposit will also significantly increase the current density compared to the unmodified recessed electrode.

Cu overpotential deposition (Figure 4b) resulted in an evenly filled hole with coral-like dendrites that are more rounded compared to the spikes seen with Au deposition, consistent with structural characteristics observed in previous studies [28,29,30]. The underpotential deposition seen in Figure 4e also gives rise to rounded surface features, and the deposit shows a similar shape and height to the Au underpotential deposits (Figure 4d). Typically, only a single monolayer of copper is formed during underpotential deposition [31]; however, a more negative potential in the kinetically controlled region was utilised to achieve the desired deposit size and growth. Further magnification revealed a rough surface for both structures, with deep grooves in the overpotential electrodeposited structure (Figure 5b,e). This is expected to result in a significant increase in the electroactive surface area.

The chronoamperometric behaviour during Pd deposition (Figure 3c) was quite different to that of Au or Cu, which may explain the different shape of the Pd overpotential deposit, which formed a coral-like structure with an obvious hole in the center. The edges of the deposit have larger growth segments likely due to the faster radial diffusion occurring to the edges compared to linear diffusion around the center of the hole, as was observed previously for Pt [12]. Further magnification in Figure 5c reveals that the surface of the structure is covered in smaller rounded structures, which will overall increase the electroactive surface area. The small nodules apparent on the surface and their rounded structure are consistent with previous Pd electrodeposition studies [24,32]. The size of the deposits were not entirely consistent across the array, with some appearing to have segments detached during rinsing, suggesting that the structures are slightly more fragile than the Pt deposits in our previous study [12]. Care was taken to wash the Pd structures after deposition. The underpotential deposits seen in Figure 4f also show very little material at the center of the structure, but have a more rounded coral-like structure compared to the overpotential deposits. Further magnification (Figure 5f) show these large rounded features have ridge-like surface details.

### 3.3. Electroactive Surface Area Calculation

To calculate and compare the sensing current density on the deposited microarrays, the electroactive surface area (ESA) was first calculated using CVs recorded in 0.5 M H_2_SO_4(aq)_. During attempts to characterise the copper deposits, it was found that in H_2_SO_4(aq)_, Ru(NH_3_)_6_Cl_3(aq)_ and K_3_[Fe(CN)_6_]_(aq)_ solutions, copper was stripped off the array within just five cycles. Therefore, conventional ESA determination was not possible for the copper deposits. On the Au and Pd electrodes, the ESA was quantified using the classical reduction of the surface oxide (Au_2_O_3_ and PdO respectively) observed in acidic media according to the following equation [33]:

(4) $$\mathrm{ESA}\;\left({\mathrm{cm}}^{2}\right)=\;\frac{Q_{H}\;\left(\mu C\right)}{x\;\left({\mu C\;\mathrm{cm}}^{-2}\right)}$$

where *x* is 390 µC cm^−2^ for Au and 410 µC cm^−2^ for Pd [33]. *Q*_H_ is the charge calculated by integrating the oxide reduction peak and is given by:

(5) $$Q_{H}=I\;\times t$$

where *I* = current and *t* = time. The calculated ESA values are shown in Table 1.

The ESA values show a modest increase from the unmodified electrodes compared to our previous study for Pt [13], which showed a ten-times increase for the cauliflower deposits. A four-times increase in ESA was observed for the Au overpotential modWEs compared to the unmodified recessed Pt MATFE (unmodWE) (see Table 1). This was similar for Pd, where an approximate five-times increase was observed.

### 3.4. Electrochemical Sensing Experiments

The modWEs were employed for the detection of different analytes in RTILs to investigate how surface modification impacts the sensing performance. Generally, the increased electroactive surface area of the modWEs (Table 1) and enhanced radial diffusion profiles should significantly enhance the sensitivity towards the respective analytes. The different nature of the electrode material is also expected to have an effect. In this study, the sensitivity enhancement from both overpotential and underpotential deposition are compared for the different metals.

Au was chosen as an electrode surface, due to its electrocatalytic properties for the reduction of nitroaromatic explosive compounds such as 2,4,6-trinitrotoluene (TNT), and has previously shown less passivation compared to other electrode surfaces [34]. Next, previous investigations of CO_2_ reduction on Cu electrodes have revealed an enhanced electrocatalytic ability compared to traditional electrodes surfaces (Pt, Au) which produce little to no current response [35]. Furthermore, Cu is commonly used for electroplating and its electrodeposition behavior is well understood. For these reasons, Cu deposits were investigated for CO_2_ reduction in analytical detection devices. Lastly, Pd is well known for its hydrogen catalytic properties, and with increasing focus on H_2_ as a fuel alternative [36,37], it may be utilised as a highly sensitive electrode surface for leak detection.

#### 3.4.1. Electrochemical Detection of TNT on Au ModWEs

**Figure 6:**
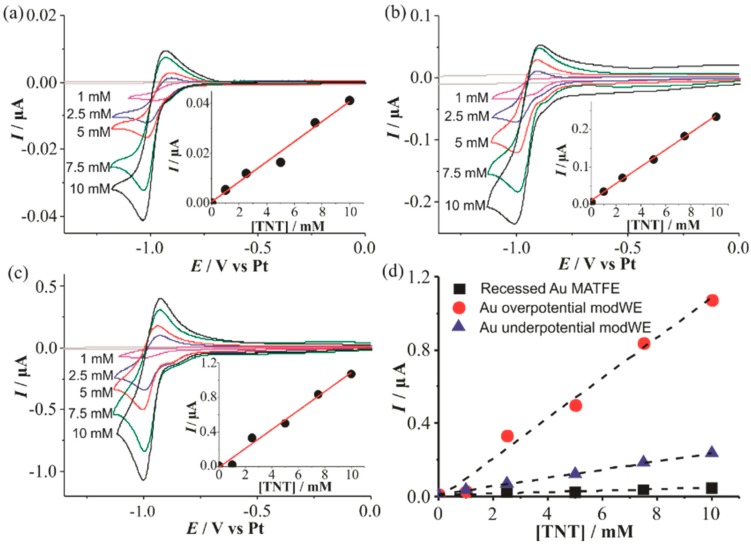
Cyclic voltammetry (CV) for the reduction of 2,4,6-trinitrotoluene (TNT) (1–10mM) in [C_4_mim] [NTf_2_] on (**a**) a recessed Au MATFE, (**b**) Au underpotential modWE and (**c**) Au overpotential modWE at a scan rate of 100 mVs^−1^. Grey line is the response in the absence of TNT. The insets show background-subtracted calibration plots of peak current vs. concentration along with the line of best fit. Plots of absolute current vs. TNT concentration are shown in (**d**) on an unmodified Au MATFE (■), Au overpotential modWE (●), and Au underpotential modWE (▲).

Figure 6 shows CVs for the reduction of 1–10 mM TNT in the RTIL [C_4_mim] [NTf_2_] on a (a) Au unmodWE, (b) underpotential Au modWE, and (c) overpotential Au modWE. The peak currents were plotted against the respective concentration, shown in the insets. All electrodes showed a response of three distinct peaks (not shown here), corresponding to the reduction of each of the three aromatic nitro-groups as reported in our previous work [38]. The first peak is chemically reversible on all three electrodes, consistent with previous research [38], and this reduction peak current is used for sensing in this work. This reduction peak corresponds to the one-electron reduction of a single aromatic nitro-group to the radical anion, and the reverse (oxidation) peak corresponds to the oxidation of the electrogenerated product [38]. The modWEs showed a larger background response due to the increased electroactive surface area, however, the background was easily subtracted from the analyte response. Calibration graphs were plotted for each electrode and linear responses were observed, with the highest linearity on the underpotential Au modWE (R^2^ > 0.998).

The sensitivity of the electrodes improved almost 30 times on the overpotential Au modWE, with the underpotential modWE improving almost 10 times, (Table 2) thus demonstrating the advantage of modification by electrodeposition. This is much larger than the electroactive surface area enhancements of ~2.5 to 4 times shown in Table 1, suggesting that increased radial diffusion is occurring to the 3D structures.

#### 3.4.2. Electrochemical Detection of Carbon Dioxide on Cu ModWEs

Prior to examining the analytical response of the modified microarrays, CV was performed to observe the voltammetric behaviour of CO_2_ reduction on the Cu electrodes. As carbon dioxide is not electrochemically active on most conventional electrode surfaces (e.g., Pt or Au), there is little prior research into the mechanism of carbon dioxide reduction in RTILs [35]. Furthermore, the possible reduction products for carbon dioxide can be quite varied depending on the solvent and electrode surface employed [39]. For example, CO_2_ reduction in RTILs on a silver electrode shows a single reduction wave corresponding to a one-electron transfer to the radical anion [35]:

(6) $${\mathrm{CO}}_{2}+e^{-}⇌{\mathrm{CO}}_{2}{}^{•-}$$

The radical can further form carbon monoxide or an oxalate ion.

(7) $$2{\mathrm{CO}}_{2}{}^{•-}\to \mathrm{CO}+{\mathrm{CO}}_{3}{}^{2-}$$

(8) $$2{\mathrm{CO}}_{2}{}^{•-}\to C_{2}O_{4}{}^{2-}$$

**Figure 7:**
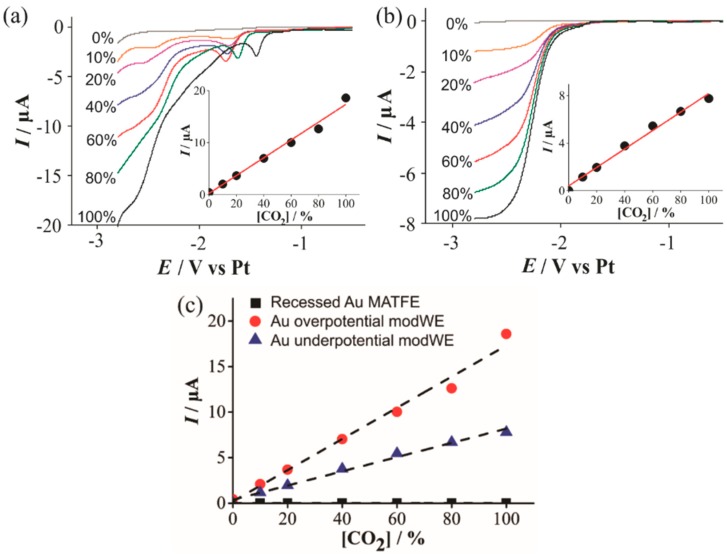
Linear sweep voltammetry (LSV) for the reduction of CO_2_ (10%–100% vol.) in [C_4_mpyrr] [NTf_2_] on a (**a**) Cu overpotential modWE and (**b**) Cu underpotential modWE at a scan rate of 100 mVs^−1^. Grey line is the response in the absence of CO_2_. The insets show background-subtracted calibration plots of peak current vs. concentration along with the line of best fit. These calibration plots are overlaid in (**c**), showing the response on a recessed Pt MATFE (■), Cu overpotential modWE (●), and Cu underpotential modWE (▲). Due to the absence of a clear peak, currents were measured at a potential of −2.7 V.

However, on a copper electrode, it can undergo reduction to yield methane, ethane, aldehydes and alcohols [39]. In our study, CO_2_ voltammetric reduction waves were absent on the unmodified Pt- and Au-MATFEs, but obvious currents were observed on the Cu modWEs. Figure 7 shows linear sweep voltammetry (LSV) for the reduction of 10%–100% CO_2_ in the RTIL [C_4_mpyrr] [NTf_2_] on an (a) overpotential Cu modWE, and an (b) underpotential Cu modWE. Steady-state-like responses are observed at potentials ~−2.5 V on both electrodes; however, a sloping behavior is present on the overpotential modWE (Figure 7a). This may likely be attributed to the different surface morphology of the deposits. A pre-peak was also observed prior to the large reduction peak, which was very obvious on the overpotential modWE (Figure 7a), but more subtle on the underpotential modWE (Figure 7b). The reasons for this feature are unclear and worthy of follow-up studies, but are out of the scope of this work. Reductive peak currents for both electrodes were measured from where the plateau was observed and were plotted against the respective concentration, as shown in the insets in Figure 7. A comparative plot with the current responses for all electrodes are shown in Figure 7c. The peak current at 100 % CO_2_, and the sensitivity of the calibration graphs on the Cu modified surfaces are shown in Table 3. As no response for CO_2_ was observed on the commercially available microarrays (black squares in Figure 7c), Cu electrodeposition presents as a cheap electrode modification technique with excellent electrocatalytic ability for CO_2_ reduction and detection.

#### 3.4.3. Electrochemical Detection of Hydrogen on Pd ModWEs

The Pd-modified arrays were used to study hydrogen oxidation at different concentrations in the RTIL [C_4_mim] [NTf_2_]. The hydrogen oxidation mechanism in RTILs containing the [NTf_2_]^−^ anion on conventional Pt electrodes generally shows a single oxidation peak corresponding to the oxidation of hydrogen to its protons (which become solvated by the anion) according to the following equation [40,41]:

(9) $$H_{2}\;⇌\;2H^{+}+e^{-}$$

**Figure 8:**
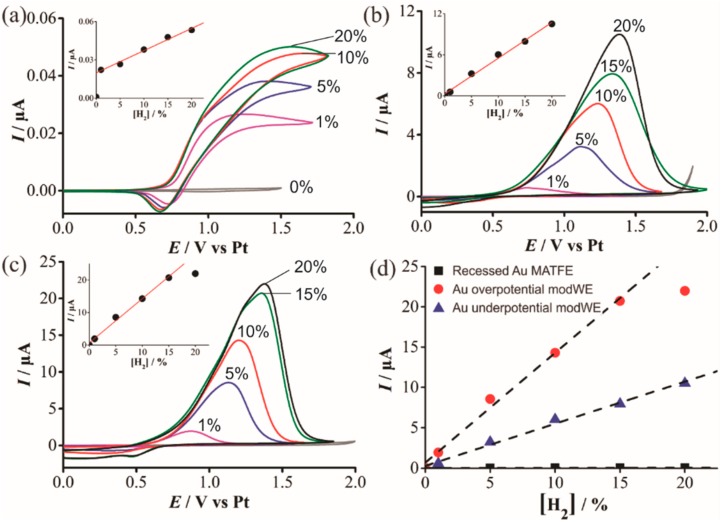
Cyclic voltammetry (CV) for the oxidation of H_2_ (1%–20% vol. gas phase) in [C_4_mim] [NTf_2_] on a (**a**) recessed Pt MATFE, (**b**) Pd overpotential modWE and (**c**) Pd underpotential modWE at a scan rate of 100 mVs^−1^. Grey line is the response in the absence of H_2_. The insets show background-subtracted calibration plots of peak current vs. concentration, along with the line of best fit. These calibration plots are overlaid in (**d**), showing the response on an unmodified Pt MATFE (■), Au overpotential modified Pt MATFE (●), and Au underpotential modified Pt MATFE (▲).

H_2_ has been shown to spontaneously adsorb on Pd to form reactive PdH_x_ [36,37] but the effect for H_2_ sensing on Pd is relatively unknown. Figure 8 shows CVs for the oxidation of H_2_ in [C_4_mim] [NTf_2_] on a (a) Pt unmodWE, (b) underpotential Pd modWE and (c) overpotential Pd modWE. The Pt unmodWE displays a broad oxidative feature with a small corresponding reductive peak (Figure 8a), whereas, a large peak is observed on both Pd modWEs (Figure 8b,c). On the Pd modWEs, the current falls almost to zero after the oxidation peak compared to a classical diffusion-controlled peak shape. This suggests that the oxidation of H_2_ on Pd is governed by a surface-confined process, involving the adsorption of H_2_ as a thin layer across the rough surface of the electrode [42]. It is noted that for a standard redox couple, hexaammineruthenium(III) chloride in 0.1 M KCl, classical peak shapes were observed on these electrodes, further ruling out that the peak-shape is due to other factors such as confinement of analyte within a porous electrode [43], and that the behaviour observed is most likely due to the adsorption of H_2_ onto the electrode surface. Just like for TNT detection on Au in Section 3.4.1, a substantial increase in current density was also observed on the Pd modWE vs. a Pt unmodWE (see Table 4). Most notably, on the overpotential Pd modWE, a more than 1000-fold increase in sensitivity towards H_2_ vs. bare Pt-MATFE is achieved.

Oxidative peak currents for all electrodes were measured and are plotted against the respective concentration in the insets of Figure 8. A calibration plot comparing the current responses for all electrodes is shown in Figure 8d. For the overpotential modWE, the current plateaued at concentrations above 15% vol. H_2_. This may be due to passivation of the electrode surface, further aggravated by the formation of PdO. The detection of hydrogen in RTILs on Pt is known to be more complicated than a typical diffusion-controlled process, due to the adsorption of H_2_ onto electrode surfaces [40,43,44]. Overall, a significantly greater current is observed on the Pd electrode and this is largely attributed to its adsorption properties towards H_2_, as well as the larger electroactive surface area. The difficulties in obtaining a linear calibration graph is consistent with previous studies of its oxidation on porous Pt electrodes [43].

## 4. Conclusions

Different metals were deposited into the microholes of MATFEs, and the resulting modWEs were used for sensing applications in ionic liquids. Gold deposition using an overpotential formed spiky structures with large dendrites, whereas underpotential depositions formed ball-like structures with finer, spiky surface details. The copper structures deposited at overpotentials were coral-like in appearance with underpotential deposits having a similar shape to gold but with more rounded surface details. The Pd structures formed “bubbly” coral-like structures with an obvious hole in the centre. The electroactive surface areas (ESA) of the modified electrodes (modWEs) were characterized with cyclic voltammetry in acidic solutions, and showed an ~5 times increase in ESA. This agrees with the unique rough fractal structures observed from scanning electron microscopy characterisation. For TNT sensing on Au modWEs, a 30-times increased sensitivity was achieved compared to the unmodified electrode. CO_2_ voltammetric electroreduction waves were clearly obtainable on the Cu modWEs, despite being absent on the bare Pt-MATFE, showing the excellent electrocatalytic activity. Lastly, for H_2_ sensing on Pd modWEs, more than 1000-fold improvement in sensitivity was achieved compared to the unmodified Pt-MATFE. Overall, we have demonstrated significantly enhanced selectivity and electrocatalytic behaviour towards analytes of interest by the formation of Au, Cu and Pd 3-D nanostructured arrays.

## Figures and Tables

**Table 1:** Deposition parameters: deposition potential, charge limit fixed for deposition, average deposition time (from three repeat deposits), charge (*Q*_H_), and electroactive surface area (ESA). *Q*_H_ and ESA were calculated from the integration of the Au_2_O_3_ or PdO reduction peak obtained in 0.5 M H_2_SO_4_ at 500 mVs^−1^ vs. Ag/AgCl.

	Modification	Deposition Potential/V	Charge Limit/mC	Average Deposition Time/s	*Q*_H_/µC	ESA/mm^2^
**Au**	Unmodified	-	-	-	3.55	0.909
	Overpotential	0.1	−1.5	55	4.33	4.10
	Underpotential	0.5	−1.5	450	1.87	2.57
**Cu**	Overpotential	−0.4	−4.5	150	-	-
	Underpotential	−0.2	−4.5	600	-	-
**Pd**	Unmodified	-	-	-	2.15	1.02
	Overpotential	0.1	−1.5	50	2.17	5.17
	Underpotential	0.35	−1.5	250	1.96	4.66

**Table 2:** Analytical parameters obtained for TNT reduction in [C_4_mim] [NTf_2_]: reduction peak current (*I*_p_) for 10 mM TNT, current density (*J*) for 10 mM TNT, and sensitivity calculated for TNT peak 1 reduction (1–10 mM).

Au Modification	*I*_p_ (10 mM TNT)/A	*J*/Am^−2^	Sensitivity/AM^−1^
Unmodified Au	−4.84 × 10^−8^	−5.32 × 10^−2^	3.89 × 10^−6^
Overpotential	−1.07 × 10^−6^	−2.61 × 10^−1^	1.10 × 10^−4^
Underpotential	−2.35 × 10^−7^	−9.14 × 10^−2^	2.26 × 10^−5^

**Table 3:** Analytical parameters obtained for CO_2_ reduction in [C_4_mpyrr] [NTf_2_]: reduction peak current (*I*_p_) for 100% CO_2_, and sensitivity calculated for CO_2_ reduction (10%–100%).

Cu Modification	*I*_p_ (100% CO_2_)/A	Sensitivity/A%vol.^−1^
Overpotential	−1.86 × 10^−5^	1.54 × 10^−7^
Underpotential	−7.79 × 10^−6^	8.35 × 10^−8^

**Table 4:** Analytical parameters obtained for H_2_ oxidation in [C_4_mim] [NTf_2_]: oxidation peak current (*I*_p_) for 15% H_2_, current density (*J*) 15% H_2_, and sensitivity calculated for H_2_ oxidation (1%–15%).

Pd Modification	*I*_p_ (15% H_2_)/A	*J*/Am^−2^	Sensitivity/A%vol.^−1^
Unmodified	4.17 × 10^−8^	4.07 × 10^−2^	1.05 × 10^−9^
Overpotential	2.07 × 10^−5^	4.01	1.36 × 10^−6^
Underpotential	7.94 × 10^−6^	1.71	5.22 × 10^−7^
